# Temperature in Phthisis

**Published:** 1902-05-17

**Authors:** 


### Temperature in Phthisis.

There are few chronic diseases in which temperature has been more carefully studied than it has in pulmonary phthisis, and it may be added there are few in which the results have been more confusing. That the occurrence of much pyrexia is a thing to be regarded with anxiety has been recognised from the early days of medical thermometry, but that in some cases the disease might make steady progress in the absence of any serious fever was equally clear, while it has been maintained that in the early stages of the malady the temperature may be actually subnormal. Notwithstanding all such discrepancies, however, there is no doubt that the general feeling of the profession has been that tuberculosis is a pyrexial disorder, and that its activity may be measured by the amount of fever with which it is attended. This is well expressed in Allbutt's "Medicine." "Pyrexia," it is said, "is a symptom hardly less significant, from the point of view of diagnosis, than cough. The cause of the elevation must be ascribed to the presence in the blood of some subtle poison produced by the bacillus." And again, "It may be stated as a general principle that activity of the disease is indicated more surely by pyrexia than by any other symptom or sign."

Further observation, however, especially observation made under what one may call natural conditions upon patients living in the open air and protected as far as possible from intercurrent catarrhal and other complications, have led to considerable modifications of the older and simpler view. It has become plain that phthisis never goes very far without the tuberculosis element becoming complicated with various other infections, and that these, and perhaps various other causes, are responsible for a good deal of the pyrexia which had formerly been put down to the tuberculous process. Thus, without denying that advancing and generalising tuberculosis is a febrile process, it has become apparent that in any given case, especially of ordinary chronic phthisis, the fever present cannot be safely taken as a measure of the activity of the primary disease, however much it may be an indication, which indeed it is, that something is going wrong with the patient. This view of the matter has been strongly enforced by some observations lately recorded by Dr. R. W. Philip[1] of Edinburgh who shows how largely success in treating phthisis depends upon the care taken to discover and eliminate this superadded "something" to which is due so much of the destructive pyrexia met with in pulmonary tuberculosis. As the result of his researches he shows that although patients when coming under observation may suffer from various degrees of fever, the majority when placed on open-air lines show a remarkably rapid return of temperature to the normal, and this notwithstanding that their physical signs may indicate considerable activity in their disease. In a certain proportion of the cases, however, the fall in temperature does not occur so rapidly. Still even if delayed for several months ultimate return to the normal is frequent if proper treatment be maintained, and it is a grievous mistake to assume, as is so often done, that if the temperature does not return to the normal in three months it will not do so at all, whatever course be followed. On the other hand, return to the normal temperature does not always mean that the lung is returning to its normal condition, for, according to Dr. Philip, the temperature may remain almost continuously normal even when abundant evidence of moisture is present and other signs suggest the presence of considerable disease in a state far from a latent. This observation is one of the highest importance, and is, of course, the key to the position taken up, namely, that the pyrexia in phthisis is often produced by other than the tuberculous processes which are going on. Thus we come to the main conclusion of the matter, namely, that when the temperature is disturbed, either continuously or recurrently, such disturbance should not be attributed vaguely to tuberculosis—a definite superadded cause of disturbance should be sought for, and if possible removed. Dr. Philip says that putting aside such definite causes of pyrexia as broncho-pneumonia or miliary dissemination of tubercle throughout the lung, the temperature may be disturbed by a great variety of causes. Thus a pleurisy or an influenza may occur, or the latter, as sometimes happens, may set up a broncho-pneumonia. At other times the cause is still simpler, such as an unhealthy condition of the teeth and gums, a condition which should always be looked for and most assiduously treated by antiseptic measures. Or the rise may be due to undue effort, as in walking, riding, and the like, which may bring on pyrexia even where the temperature had previously been continuously normal. Nervous irritation, worry, or the receipt of bad news may also send up the thermometer. If Dr. Philip's observations are to be accepted as correct, tubercle *per se* plays a minor part in the production of the pyrexia with which pulmonary tuberculosis is so often associated. But it always shows that something is going wrong which must be discovered and relieved if the major disease is to obtain the full benefit of treatment.

[1] Practitioner, May 1902.

